# Exploring the mechanism of Suanzaoren decoction in treatment of insomnia based on network pharmacology and molecular docking

**DOI:** 10.3389/fphar.2023.1145532

**Published:** 2023-08-21

**Authors:** Shuxiao Wang, Yan Zhao, Xingang Hu

**Affiliations:** ^1^ Internal Encephalopathy of Traditonal Chinese Medicine, Beijing University of Chinese Medicine Third Affiliated Hospital, Beijing, China; ^2^ School of Traditional Chinese Medicine, Beijing University of Chinese Medicine, Beijing, China; ^3^ Internal Encephalopathy of Traditonal Chinese Medicine, Dongfang Hospital Beijing University of Chinese Medicine, Beijing, China

**Keywords:** Suanzaoren decoction, insomnia, network pharmacology, molecular docking, mechanism

## Abstract

**Objective:** To explore the functional mechanisms of Suanzaoren decoction (SZRD) for treating insomnia using network pharmacology and molecular docking.

**Methods:** The active ingredients and corresponding targets of SZRD were obtained from the Traditional Chinese Medicine Systems Pharmacology database, and then, the names of the target proteins were standardized using the UniProt database. The insomnia-related targets were obtained from the GeneCards, DisGeNET, and DrugBank databases. Next, a Venn diagram comprising the drug and disease targets was created, and the intersecting targets were used to draw the active ingredient-target network diagram using Cytoscape software. Next, the STRING database was used to build a protein-protein interaction network, followed by cluster analysis using the MCODE plug-in. The Database for Annotation, Visualization, Integrated Discovery (i.e., DAVID), and the Metascape database were used for Gene Ontology (GO) enrichment and Kyoto Encyclopedia of Genes and Genomes (KEGG) pathway analyses. AutoDock Vina and Pymol software were used for molecular docking.

**Results:** SZRD contained 138 active ingredients, corresponding to 239 targets. We also identified 2,062 insomnia-related targets, among which, 95 drug and disease targets intersected. The GO analysis identified 490, 62, and 114 genes related to biological processes, cellular components, and molecular functions, respectively. Lipid and atherosclerosis, chemical carcinogen-receptor activation, and neuroactive ligand-receptor interaction were the most common pathways in the KEGG analysis. Molecular docking demonstrated that the primary active components of SZRD for insomnia had good binding capabilities with the core proteins in PPI network.

**Conclusion:** Insomnia treatment with SZRD involves multiple targets and signaling pathways, which may improve insomnia by reducing inflammation, regulating neurotransmitters.

## 1 Introduction

Insomnia is a common sleep disorder primarily manifesting as difficulty falling asleep, decreased sleep quality, and shortened sleep duration (Punnoose et al., 2012). The annual incidence of insomnia has been on a rise with developing social economy, quick pace of life, and increased societal pressure. For instance, the overall global incidence rate of sleep-wake disorders is 27% (Wickwire et al., 2016) across all age groups. However, the prevalence rates vary from children to older adults, with an annual prevalence of ∼5% and a prevalence of ∼50% in patients with chronic insomnia over a 1- to 20-year follow-up period, occurring most frequently in high-income countries (Paul et al., 2022). Insomnia affects daily life, such as study and work, and is an independent risk factor for coronary heart disease, acute myocardial infarction, heart failure, hypertension, diabetes, and other chronic diseases (Wei et al., 2019). Long-term insomnia can also induce anxiety, depression, and other mental disorders (Hertenstein et al., 2019; Chellappa and Aeschbach, 2022).

Many research groups have investigated the mechanisms involved in the prevention and treatment of insomnia, however, there are several causes, and the mechanisms are complex. Insomnia-related mechanistic studies have explored the hypothalamic-pituitary-adrenalin axis dysfunction, vagal tone changes, melatonin system function decline, the influence of inflammatory response factors, central neurotransmitter disorders, and functional or structural abnormalities of the limbic cortical system circuits (Cheng et al., 2016). In addition, few medications are available to treat insomnia including benzodiazepine receptor agonists (BZRAS), melatonin receptor agonists (MRA), antidepressants, and orexin receptor antagonists (Wei et al., 2019), of which BZRAS and MRA are the most commonly prescribed (Liu et al., 2019). These medications work quickly and deliver considerable relief, but they are accompanied by adverse effects, such as dry mouth and drowsiness; thus, they should be taken only for a limited period (Shang et al., 2021).

Traditional Chinese medicines (TCM), such as Suanzaoren decoction (SZRD), are safe and effective treatments for insomnia. Wang et al. (2022) conducted a 300-person clinical study of insomnia and reported that SZRD improved sleep quality and anxiety and depression symptoms. SZRD was written in the Synopsis of the Golden Chamber by Zhongjing Zhang in the Eastern Han Dynasty and is a common treatment for insomnia today. Moreover, SZRD is often modified for treating other syndromes; for instances. Suanzaoren nourishes the liver and calms the heart and mind. Moreover, its pharmacological active ingredient, Jujube saponin A, has sedative effects, improves sleep, protects the nervous system, improves memory, and elicits antioxidant and anti-inflammatory effects (Yang et al., 2023). However, the results of these pharmacological effects are preliminary and require validation, and the specific insomnia treatment-related mechanisms of SZRD remain unclear.

SZRD is prepared with Suanzaoren, Chuanxiong, Gancao, Fuling, and Zhimu. Ligustrin I, the active component of Chuanxiong, penetrates the blood-brain barrier (Xiong et al., 2013), regulates monoamine neurotransmitters, and reduces the nitric oxide concentration in the brain and blood (Xiong et al., 2013). In addition, Poria cocos polysaccharide, the active ingredient of Fuling, has calming, anti-inflammatory, and antioxidant effects (Wang et al., 2011), and licorice extract reduces oxidative stress, decreases nerve cell damage, and protects nerves (Deng et al., 2021).

TCM prescriptions comprise several components that exert their effects via several pathways, requiring multi-target coordination. Therefore, a comprehensive understanding of the pharmacodynamic material basis and mechanisms is essential. The multi-layer and multi-angle research strategy of network pharmacology coincides with the systematic and holistic view of TCM. Therefore, in this study, we used bioinformatics to predict SZRD-specific therapeutic targets and signaling pathways and explore potential insomnia-related mechanisms.

## 2 Materials and methods

### 2.1 Identifying SZRD targets

Active ingredients and the corresponding targets of the single-flavor Chinese medicines in SZRD (Suanzaoren, Chuanxiong, Zhimu, Gancao, and Fuling) were identified using the Traditional Chinese Medicine Systems Pharmacology Database and Analysis Platform (TCMSP; https://old.tcmsp-e.com/tcmsp.php) (Ru et al., 2014), filtered by an oral bioavailability of ≥30% and a drug-likeness of ≥0.18 (Lin et al., 2021). The active ingredient targets were converted into standard gene names using the Uniprot database (https://www.uniprot.org, UniProt Consortium, 2023).

### 2.2 Insomnia-related mechanistic targets

The keyword “Insomnia” was searched in the GeneCards database (https://www.genecards.org) (Stelzer et al., 2016), DisGeNET database (https://www.disgenet.org) (Pinero et al., 2021), and DrugBank database (https://www.drugbank.com) (Wishart et al., 2018) to identify insomnia-related gene targets; the results of the three databases were integrated.

### 2.3 Mapping the active ingredient-target network

We integrated the active ingredient (drug)-related and disease-related targets to identify overlapping targets. The active ingredient-target network was generated using Cytoscape 3.9.1 software; the nodes represent the active ingredients and targets, and the edges represent the interrelationships between them. The Network Analyzer is a built-in tool to calculate the degree value, which was used to screen the major active ingredients.

### 2.4 Constructing protein-protein interaction (PPI) networks

The intersection targets were uploaded to the STRING database (https://cn.string-db.org) (Szklarczyk et al., 2019) to construct the PPI network; the organism was set to “*homo sapiens*” with an interaction score of ≥0.4. The PPI network data was uploaded to Cytoscape to obtain the core proteins with their standard degree values, closeness centrality (CC), betweenness centrality (BC), and neighborhood centrality (NC). Then, they were analyzed using the molecular complex detection (MCODE) plug-in.

### 2.5 Gene Ontology (GO) and Kyoto Encyclopedia of Genes and Genomes (KEGG) pathway enrichment analyses

A GO analysis of the intersection targets was performed using the Database for Annotation, Visualization, and Integrated Discovery (i.e., DAVID; https://david.ncifcrf.gov) (Huang et al., 2009). GO analysis results comprise genes related to biological processes (BP), cellular components (CC), and molecular functions (MF); the results were screened for *p*-values of <0.05. The KEGG pathway enrichment analysis was performed using the Metascape database (https://metascape.org) (Zhou et al., 2019), and 20 pathways with a *p*-value of <0.05 were selected and sorted by *p*-value from smallest to largest.

### 2.6 Molecular docking

Molecular docking was performed using the active ingredients with the highest degree value in the active ingredient-target network and the core targets in the PPI network. The two-dimensional structures of the small-molecule ligands of the active ingredients were obtained from the PubChem database (https://pubchem.ncbi.nlm.nih.gov) (Kim et al., 2023) and then converted into three-dimensional structures using Chem3D software. Protein structures were obtained through the Protein Data Bank database (https://www.rcsb.org) (Fermi et al., 1984). Water molecules and small molecule ligands were removed from the protein structures using Pymol software (http://www.pymol.org/pymol), and pre-processing, such as hydrogenation and identification of active pockets, was performed using AutoDock Tools (https://ccsb.scripps.edu/autodock/). Molecular docking was performed using AutoDock Vina software (Trott and Olson, 2010), and the results were optimized for output using Pymol software.

## 3 Results

### 3.1 SZRD target integration

In total, 138 active ingredients were identified in the TCMSP platform: 9 for Suanzaoren, 7 for Chuanxiong, 92 for Gancao, 15 for Fuling, and 15 for Zhimu (Table 1). Additionally, 2,059 potential targets were identified. Finally, 239 target genes were obtained after the name transformation and de-duplication processes.

**TABLE 1:** Statistical information of “drug-active ingredient-target” of Suanzaoren decoction.

No.	Drug	Amount of active ingredient	Amount of target
1	Suanzaoren	9	46
2	Chuanxiong	7	32
3	Zhimu	15	188
4	Gancao	92	1766
5	Fuling	15	27

### 3.2 Screening insomnia-related target genes

**FIGURE 1:**
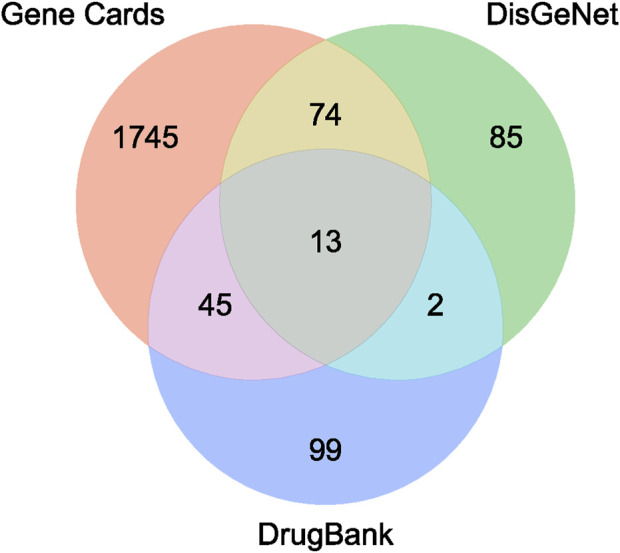
Venny diagram of insomnia targets from different database.

**FIGURE 2:**
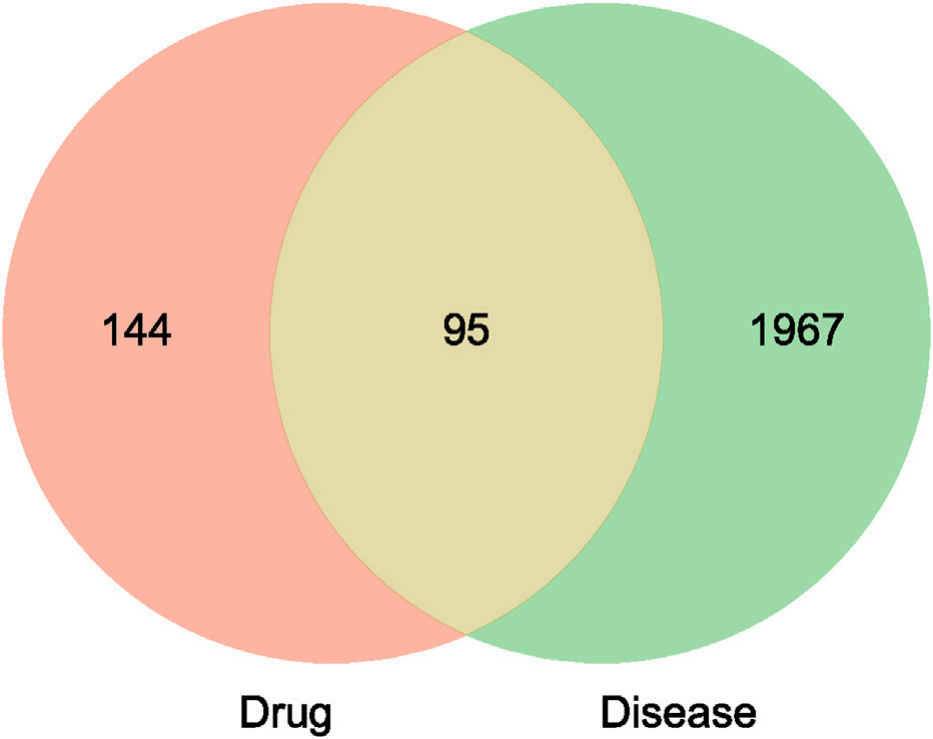
Venny diagram of active ingredient targets of Suanzaoren decoction and insomnia targets.

Figure 1 presents the insomnia-related targets identified through various databases. The GeneCards, DisGeNET, and DrugBank databases contained 1,877, 174, and 159 disease targets, respectively. Finally, 2,062 disease genes remained after removing the duplicate genes. The Venn diagram indicated that 95 disease and active ingredient targets overlapped (Figure 2).

### 3.3 Active ingredient-target network analysis

**FIGURE 3:**
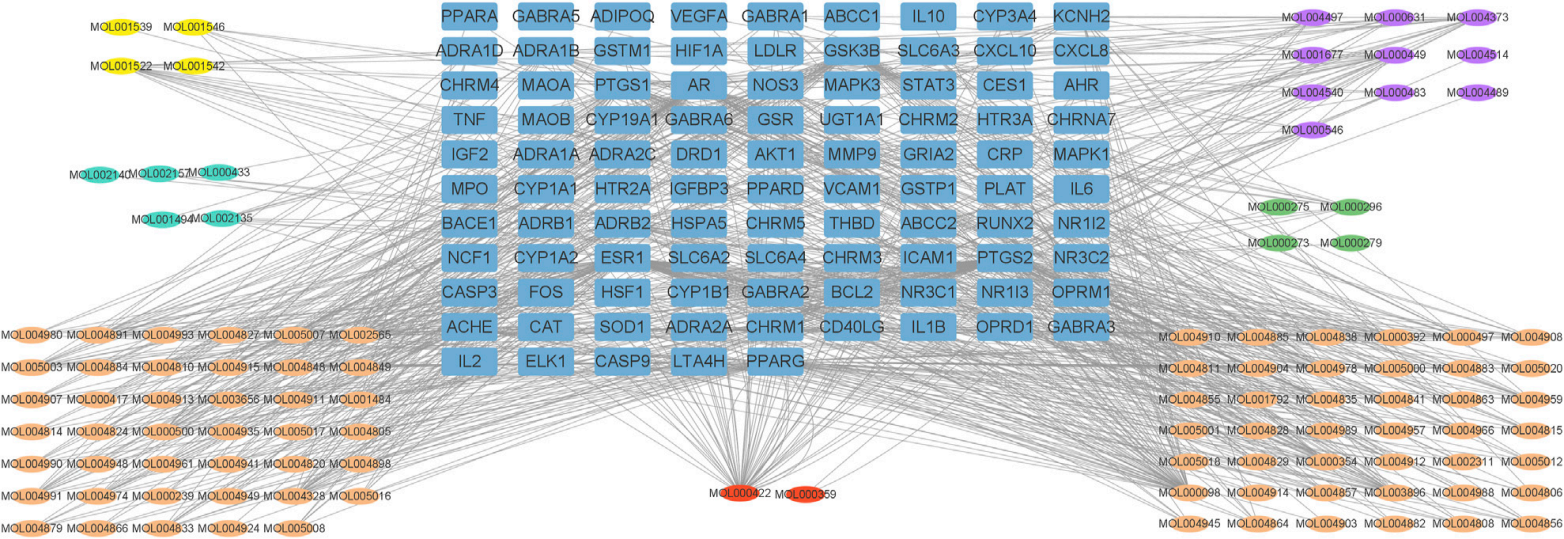
“Active ingredient-target” network of Suanzaoren decoction. Blue squares represent targets. Yellow, purple, green, orange and blue ovals represent the active ingredients from Suanzaoren, Zhimu, Fuling, Gancao and Chuanxiong, red ovals represent the active ingredients common to both Gancao and Zhimu.

The active ingredients and the 95 overlapping targets were used to construct the active ingredient-target network (Figure 3), which contained 208 nodes (113 active ingredient nodes [oval nodes of various colors] and 95 target nodes [square nodes]) and 1,614 edges (representing the interactions between the ingredients and targets). The network topology parameters for treating insomnia were analyzed using Network Analyzer in Cytoscape. The average degree value was 15.5.

The active ingredients were screened based on their degree values, and eight compounds had a degree value greater than two times the average: kaempferol, quercetin, 7-Methoxy-2-methyl isoflavone, naringenin stigmasterol, medicarpin, formononetin, and shinpterocarpin (Table 2). The degree value of kaempferol, a common active ingredient in Gancao and Zhimu, was particularly high.

**TABLE 2:** Information of top 8 active ingredients with degree value in “Active ingredient-target” network of Suanzaoren decoction.

Mol ID	Active ingredient	Dgree value	Drug
MOL000422	kaempferol	116	Gancao, Zhimu
MOL000098	quercetin	110	Gancao
MOL003896	7-Methoxy-2-methyl isoflavone	46	Gancao
MOL004328	naringenin	40	Gancao
MOL000449	Stigmasterol	40	Zhimu
MOL002565	Medicarpin	38	Gancao
MOL000392	formononetin	32	Gancao
MOL004891	shinpterocarpin	32	Gancao

### 3.4 PPI construction and analysis

**FIGURE 4:**
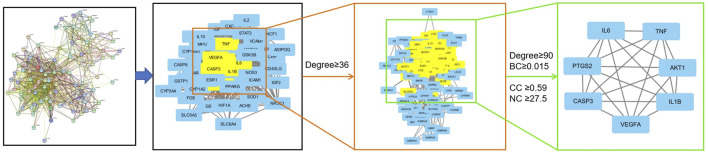
Topological screening process of the PPI network. A total of 95 common targets were screened using degree value, betweenness centrality (BC), and closeness centrality (CC), neighborhood centrality (NC), and seven core targets were obtained.

The 95 overlapping targets were uploaded to the STRING database to construct the PPI network and obtain the corresponding network data (Supplementary Table S1), which was then imported into Cytoscape for analysis. The data were screened based on degree values greater than or equal to the standard degree value and the BC, CC, and NC genes. We identified seven core targets: interleukin (IL)-6, AKT serine/threonine kinase 1 (AKT1), tumor necrosis factor (TNF), IL-1β, vascular endothelial growth factor A (VEGFA), prostaglandin-endoperoxide synthase 2 (i.e., PTGS2), and caspase 3 (i.e., CASP3) (Table 3; Figure 4). Thus, these proteins interacted strongly with other proteins and played key roles in the PPI network.

**TABLE 3:** Core target information of Suanzaoren decoction in the treatment of insomnia.

No.	Gene names	Uniprot ID	Protein names	Degree value
1	IL6	P05231	Interleukin-6	110
2	AKT1	P31749	RAC-alpha serine/threonine-protein kinase	110
3	TNF	P01375	Tumor necrosis factor	106
4	IL1B	P01584	Interleukin-1 beta	96
5	VEGFA	P15692	Vascular endothelial growth factor A	90
6	PTGS2	P35354	Prostaglandin G/H synthase 2	90
7	CASP3	P42574	Caspase-3	90

MCODE is one of the most widely used algorithms for mining protein complexes by using the intrinsic relationships of proteins in the network to find protein clusters. Further analysis of the PPI network with MCODE yielded seven protein clusters, then a GO enrichment analysis of the seven protein clusters was performed (Table 4).

**TABLE 4:** Cluster protein groups and its GO enrichment analysis.

No.	Proteins	BP
1	CAT, PPARG, PPARA, ICAM1, IL6, STAT3, CXCL10, MAPK1, CASP3, MAPK3, PTGS2, MMP9, CRP, RUNX2, NCF1, CD40LG, IL10, IL2, FOS, VEGFA, MPO, ADIPOQ, ESR1, HIF1A, CXCL8, AKT1, CASP9, IL1B, NOS3, TNF, VCAM1	Aging, positive regulation of gene expression, positive regulation of transcription, positive regulation of transcription from RNA polymerase II promoter, inflammatory response
2	CYP1A2, CYP1B1, UGT1A1, GSTM1, CYP1A1, NR1I2, GSTP1	xenobiotic metabolic process, estrogen metabolic process, steroid metabolic process, omega-hydroxylase P450 pathway, epoxygenase P450 pathway
3	AR, NR3C1, IGF2, CYP19A1, GSK3B, HSPA5, IGFBP3	positive regulation of MAPK cascade, prostate gland growth, ER overload response, intracellular steroid hormone receptor signaling pathway, positive regulation of insulin-like growth factor receptor signaling pathway
4	GABRA3, GABRA6, GABRA5, GRIA2	ion transmembrane transport, chemical synaptic transmission, synaptic transmission, gamma-aminobutyric acid signaling pathway, regulation of postsynaptic membrane potential
5	MAOB, GABRA1, CHRM2, OPRM1, HTR2A, GABRA2, HTR3A	chemical synaptic transmission, neurological system process, G-protein coupled receptor signaling pathway, regulation of membrane potential, ion transmembrane transport
6	ABCC2, CYP3A4, CES1, NR1I3	xenobiotic catabolic process, cholesterol metabolic process, xenobiotic metabolic process
7	OPRD1, ADRA2C, SLC6A3, CHRNA7	regulation of sensory perception of pain, response to nicotine, positive regulation of MAPK cascade

### 3.5 GO enrichment analysis

**FIGURE 5:**
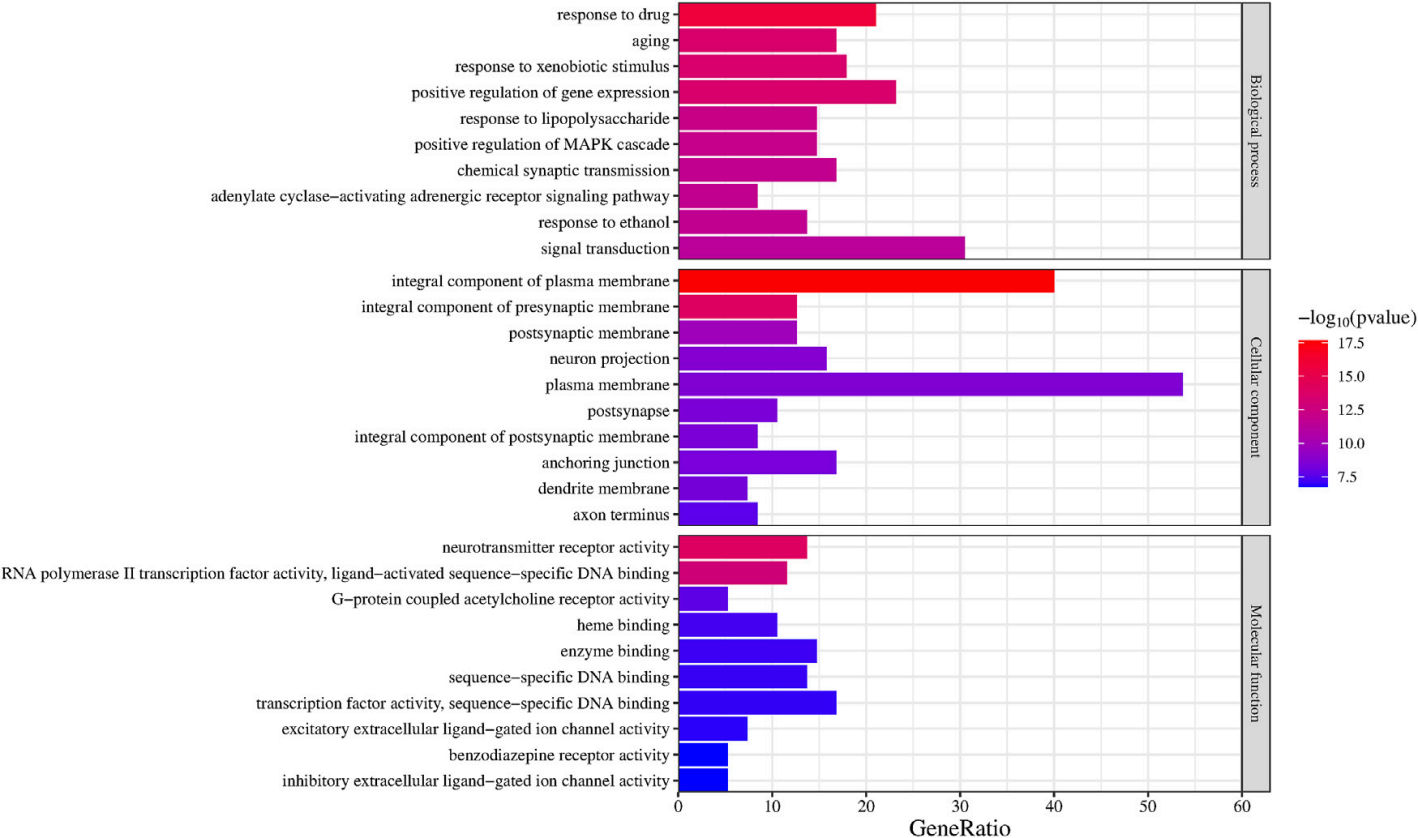
GO biological function enrichment of Suanzaoren decoction in the treatment of insomnia.

We obtained 490 BP, 62 CC, and 114 MF genes, then selected the top 10 entries of each cluster to create a bar chart based on the *p*-value (Figure 5). BPs were mainly related to drug responses, aging, responses to xenobiotic stimulus, and positive regulation of gene expression. CCs were mainly related to the integral components of the plasma membrane, the integral components of the presynaptic membrane, the postsynaptic membrane, and neuron projection. MFs primarily corresponded to neurotransmitter receptor activity, RNA polymerase II transcription factor activity, ligand-activated sequence-specific DNA binding, G-protein coupled acetylcholine receptor activity, and heme binding.

### 3.6 KEGG pathway enrichment analysis

**FIGURE 6:**
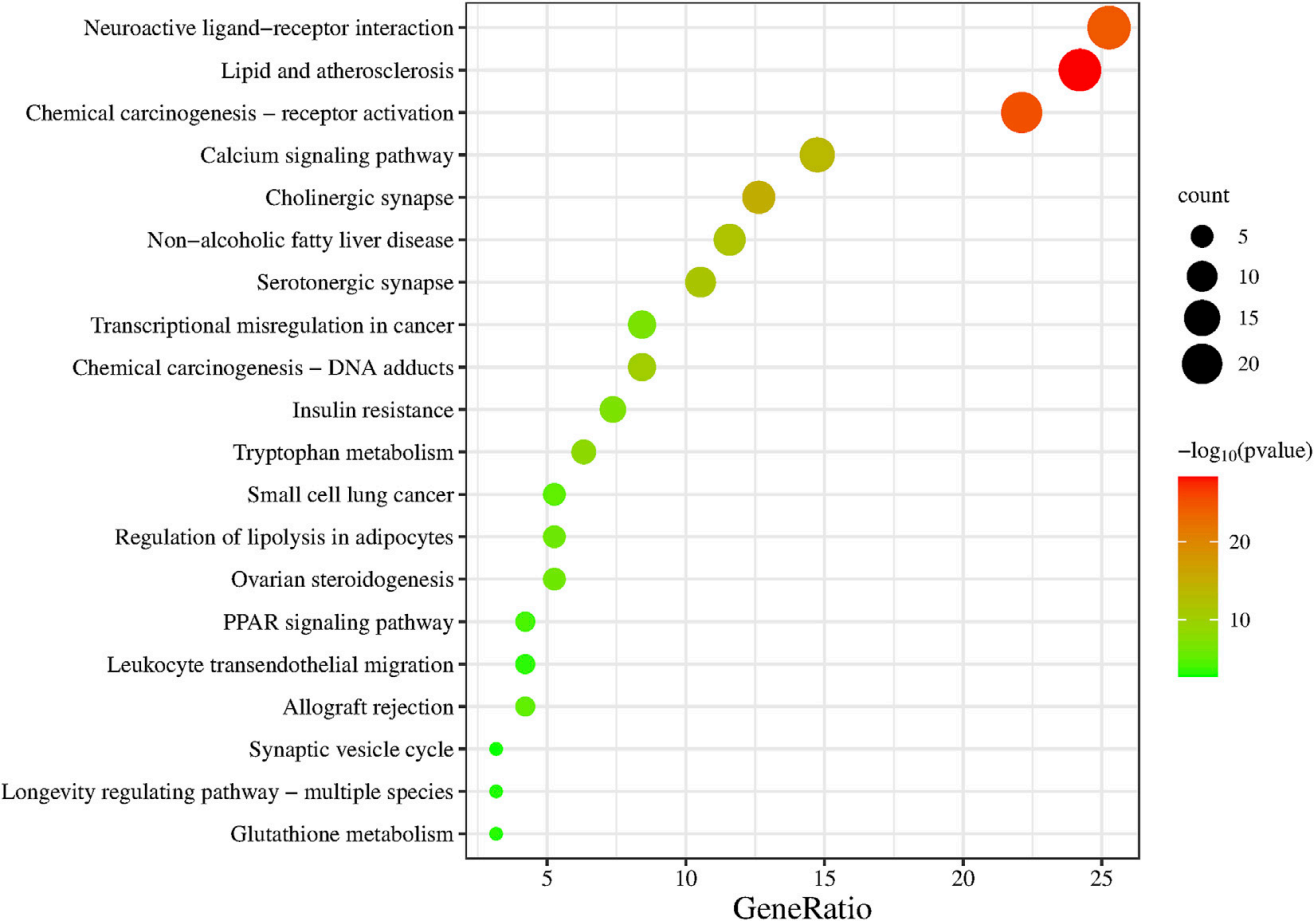
KEGG signaling pathway enrichment of Suanzaoren decoction in the treatment of insomnia. The larger the size of the dot, the more the genes are annotated in the entry, and the redder the color of the dot, the lower the q value.

**FIGURE 7:**
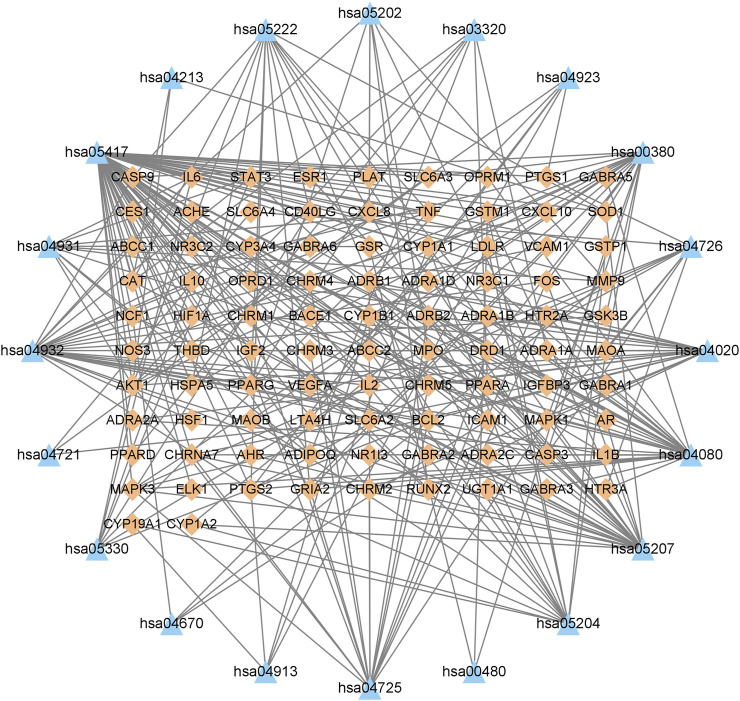
Gene–pathway network of Suanzaoren decoction against insomnia. This network shows the relationship between the enriched 20 pathways and genes. The orange diamond-shape represent target genes, and the blue triangle represent pathways.

The KEGG enrichment analysis of the 95 overlapping targets resulted in 183 signaling pathways (Supplementary Table S2). After screening based on the gene ratio and *p*-values (Figure 6), the 20 enriched summary signal pathways primarily involved lipid and atherosclerosis, chemical carcinogenesis-receptor activation, and neuroactive ligand-receptor interaction (Figure 7).

### 3.7 Molecular docking

**FIGURE 8:**
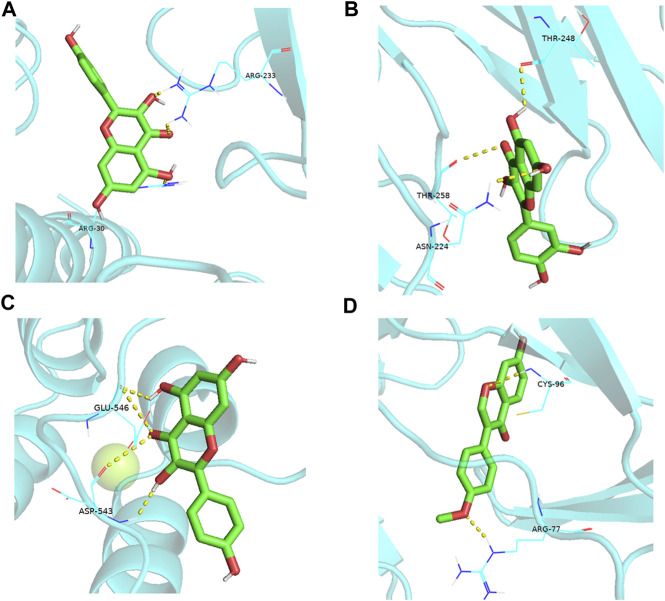
Partial diagram of molecular docking. **(A)**: IL6-kaempferol; **(B)**: IL6-quercetin; **(C)**: AKT1-kaempferol; **(D)**: TNF-formononetin.

Molecular docking is a theoretical simulation method to study the interactions between ligands and receptors and predict their binding forces and modes. Generally, the lower the energy required for receptor-ligand binding, the easier it is for the docking to succeed. Molecular docking was performed using the top nine active ingredients with a degree value of ≥30 from the active ingredient-target network (kaempferol, quercetin, 7-Methoxy-2-methyl isoflavone, naringenin, stigmasterol, and (S)-coclaurine) and the five core proteins from the PPI network (IL6, AKT1, TNF, IL-1β, VEGFA). The binding energy of the nine active ingredients to the five core proteins was <5 kcal-mol^–1^ (Table 5; Figure 8).

**TABLE 5:** Binding energy of active ingredients to target proteins (kcal·mol-1).

Mol ID	Active ingredient	IL6	AKT1	TNF	IL1B	VEGFA
MOL000422	kaempferol	−7.2	−6.9	−7.8	−6.5	−6.8
MOL000098	quercetin	−7.3	−6.9	−8.4	−6.7	−7.0
MOL003896	7-Methoxy-2-methyl isoflavone	−7.5	−7	−7.5	−5.7	−6.4
MOL004328	naringenin	−7.5	−6.6	−8	−6.5	−6.7
MOL000449	Stigmasterol	−7.3	−6.5	−7.2	−6.8	−6.8
MOL002565	Medicarpin	−7.3	−7	−8.2	−6.1	−6.3
MOL000392	formononetin	−7.5	−6.3	−8.8	−6.7	−6.7
MOL004891	shinpterocarpin	−7.5	−7.2	−7.8	−6.6	−7.2
MOL001522	(S)-Coclaurine	−7.3	−6.6	−7.3	−5.4	−5.8

## 4 Discussion

Insomnia is a common sleep disorder that is believed to be mainly caused by HPA axis dysfunction, central neurotransmitters disorders, inflammatory response factors, and changes in vagal tone. This study explored the potential of SZRD in treating insomnia using network pharmacology and molecular docking methods.

The KEGG enrichment results revealed various disease pathways unrelated to our study, probably due to shared molecular targets contributing to different diseases. Therefore, we chose to analyze the signaling pathways closely related to insomnia in the enrichment results. Our findings suggest that SZRD may exert its therapeutic effect on insomnia by modulating the neuroactive ligand-receptor interaction, serotonergic synapse, and PI3K/AKT pathway. The neuroactive ligand-receptor interaction pathway, which involves receptors and ligands on the plasma membrane associated with intra- and extracellular signaling pathways, is physiologically relevant to neural function. (Lauss et al., 2007). The neuroactive ligand-receptor interaction pathway includes 211 genes with receptors classified into four classes: class A (rhodopsin like amine), class B (secretin like), class C (metabotropic glutamate/pheromone), and channels/other receptors, (Su et al., 2009). Among these, receptors and ligands for DA, 5-HT, orexins, GABA are closely associated with sleep. The neuroactive ligand-receptor interaction pathway includes genes encoding biogenic amine receptors of subclasses such as DA and 5-HT. Dopamine (DA), an important endogenous catecholamine, plays a crucial role in the waking state. Dopamine receptors are a family of seven transmembrane regions (7-GM) comprising five subtypes of G protein-coupled receptors, of which the D1 and D5 subtypes are D1-like receptors. These D1-like receptors can be regulated to participate in the sleep-wake process by elevating intracellular cyclic adenosine monophosphate (cAMP), which acts as a second messenger to phosphorylate target cellular proteins via activating PKA (cAMP-dependent protein kinase) (Najafian et al., 2011). D2, D3, and D4 isoforms act as D2-like receptors and aid in maintaining arousal by decreasing intracellular cAMP (Najafian et al., 2011).

The orexin belongs to class A peptide subclass in the neuroactive ligand-receptor interaction signaling pathway. The orexin increases neuronal excitability by raising intracellular calcium levels through the classical phospholipase C cascade (PLC-IP2/DAG) and activates ERK 1/2 expression through Gq/PLC/PKC signaling. Orexins act through the activation of 2 G protein-coupled receptors (GPCRs), orexin receptor 1 (OX1R), and orexin receptor 2 (OX2R) (Sakurai et al., 1998). OX2R is a key receptor involved in the regulation of arousal and non-rapid eye movement (NREM) sleep. In contrast, OX1R, which primarily regulates emotional and addictive pathways, has negligible effects on sleep but acts synergistically with OX2R in regulating REM sleep (Han et al., 2020). There are three types of GABA receptors: GABAA, GABAB, and GABAC. In terms of sleep, GABAA receptor is most important. The central GABAA (γ-aminobutyric acid A) receptor is a ligand-gated ion channel receptor distributed throughout the CNS and mediates most of the inhibitory neurotransmission in the CNS. When GABA or other agonists bind to GABAA receptors, they trigger the influx of chloride ions into neuronal cells, resulting in a negative membrane potential that inhibits action potential firing; thus, GABA reduces brain cell activity by activating GABAA receptors (Kim et al., 2019).

Serotonergic synapse includes 5-hydroxytryptamine, a range of 5-hydroxytryptamine receptors, and post-receptor signaling pathways. 5-Hydroxytryptamine (5-HT) is an indole derivative catalyzed by tryptophan hydroxylase to produce 5-hydroxytryptophan, and then catalyzed by 5-hydroxytryptophan decarboxylase to generate 5-hydroxytryptophan, one of the most important neurotransmitters for sleep regulation with the highest levels in the cerebral cortex and synapses. Binding of 5-HT to its receptor activates genes associated with downstream signaling pathways, which in turn regulate sleep (Yao et al., 2021). Among the seven known receptors, the 5-HT1a and 5-HT2a receptors have highest relevance to anti-insomnia research, and cAMP is a downstream key signaling molecule acting on the post-receptor signaling pathway of 5-HT1A and 5-HT2A. 5-HT1A couples to Gαi/o (G-protein αi/o subunit), thereby dual-regulating Adenylate cyclase (AC) activity, which in turn regulates the amount of cyclic adenosine monophosphate (cAMP), which affects the brain’s regulation of mood, arousal, and circadian rhythms. 5-HT2A couples to Gαq/11 and activates PLC. PLCβ (β-Phospholipase C) is the major isoform regulated by Gαq/11 and plays an important role in the regulation of sleep and wakefulness (Luo et al., 2020).

The PI3K/Akt signaling pathway regulates the normal activity of neuronal cells, and elevated levels of phosphatidylinositol 3-kinase (PI3K) and protein kinase B (Akt) modulate the activity of genes involved in the transduction of inflammatory cytokines driving insomnia pathogenesis (Meng et al., 2013). The PI3K/Akt signaling pathway regulates neuronal cell proliferation, apoptosis, and differentiation in the brain, thereby inhibiting cell hypermobility (Xing et al., 2020) and participating in the repair of neurons and recovery of cognitive functions after sleep deprivation; this is achieved by regulating apoptosis and inflammation (Zeng et al., 2019; Wan et al., 2022). Inhibition of PI3K and Akt protein expression triggers severe sleep deprivation in rats, affecting their circadian rhythms (Huang et al., 2018), which in turn damages neuronal cells and affects learning memory functions. (Li et al., 2022). On the other hand, activation of PI3K and Akt protein expression inhibits excessive autophagy and apoptosis of neurons (Cao et al., 2021), reduces sleep deprivation-induced neuronal apoptosis (Zhou et al., 2022), and improves sleep condition (Zhang et al., 2021; Zhang et al., 2022).

The PPI network identified IL-6, AKT1, TNF, IL-1β, and VEGFA as the key proteins. IL-6, IL-1β, and TNF-α are common inflammatory factors, and significantly higher IL-6 and TNF-α levels are reported in patients with insomnia than in healthy individuals, whereas decreasing their levels had a calming effect (Burgos et al., 2006; Motivala, 2011; Xia et al., 2013). The Nod-like receptor 3 (NLRP3) inflammatory vesicle pathway is a central point for initiating and maintaining neuroinflammation and is closely associated with various neurological diseases (Hung et al., 2020). The NLRP3 inflammatory vesicle pathway promotes the maturation and secretion of the inflammatory factor IL-1β, which induces an exacerbated neuroinflammatory response (Beyer et al., 2020). IL-1β and TNF-α are downstream effector molecules of PI3K-AKT pathway (Lu et al., 2014). PI3K-AKT alters synaptic structure and function through associations and collaborations with downstream pathway proteins (Levenga et al., 2017). VEGFA exerts a neurotrophic and protective effect on the central nervous system by stimulating the release of brain-derived neurotrophic factor (i.e., BDNF) from endothelial cells (Sondell et al., 1999; Sun et al., 2003). Proteins in the PPI network also included the dopaminergic system (such as DRD1, SLC6A2, SLC6A3, SLC6A4, MAOA, and MAOB) and serotonergic system (such as HTR2A and HTR3A), whose mechanisms of action are closely related to the transmission of monoamine neurotransmitters, most of which are reportedly associated with insomnia.

In summary, our results showed that the active ingredients in SZRD bind to specific proteins, which regulate certain signaling pathways and affect downstream processes, such as the inflammatory response and neurotransmitter regulation, thereby relieving insomnia. In addition, we confirmed the multi-target, multi-pathway, and synergistic involvement of SZRD for insomnia treatment. However, TCM ingredients are complex, and the network pharmacology is limited to theoretical discussions. Therefore, further clinical and laboratory experiments are needed to validate the results. Nevertheless, our results lay a strong foundation for subsequent in-depth studies.

## Data Availability

The datasets presented in this study can be found in online repositories. The names of the repository/repositories and accession number(s) can be found in the article/Supplementary Material.

## References

[B2] Beyer M. M. S. Lonnemann N. Remus A. Latz E. Heneka M. T. Korte M. (2020). Enduring Changes in Neuronal Function upon systemic inflammation are NLRP3 inflammasome dependent. J. Neurosci. 40 (28), 5480–5494. 10.1523/JNEUROSCI.0200-20.2020 32499379 PMC7343321

[B3] Burgos I. Richter L. Klein T. Fiebich B. Feige B. Lieb K. (2006). Increased nocturnal interleukin-6 excretion in patients with primary insomnia: a pilot study. Brain, Behav. Immun. 20 (3), 246–253. 10.1016/j.bbi.2005.06.007 16084689

[B4] Cao Y. Li Q. Zhou A. Ke Z. Chen S. Li M. (2021). Notoginsenoside R1 reverses abnormal autophagy in hippocampal neurons of mice with sleep deprivation through melatonin receptor 1A. Front. Pharmacol. 12, 719313. 10.3389/fphar.2021.719313 34603030 PMC8481657

[B5] Chellappa S. L. Aeschbach D. (2022). Sleep and anxiety: from mechanisms to interventions. Sleep. Med. Rev. 61, 101583. 10.1016/j.smrv.2021.101583 34979437

[B6] Cheng G. L. Qian Y. F. Li J. Song K. K. Jiang X. W. (2016). Review about the mechanism of insomnia. World J. Sleep Med. 3 (03), 174–179.

[B7] Deng T. Peng C. Peng D. Yu X. Chen W. Wang L. (2021). Research progress on medichal constituents and pharmacological effects of Glycyrrhizae Radix et Rhizoma and discussion of Q-markers. China J. Chin. Materia Medica 46 (11), 2660–2676. 10.19540/j.cnki.cjcmm.20210304.201 34296562

[B8] Dou W. Zhang J. Sun A. Zhang E. Ding L. Mukherjee S. (2013). Protective effect of naringenin against experimental colitis via suppression of Toll-like receptor 4/NF-κB signalling. Br. J. Nutr. 110 (4), 599–608. 10.1017/S0007114512005594 23506745 PMC3726555

[B9] Fermi G. Perutz M. F. Shaanan B. Fourme R. (1984). The crystal structure of human deoxyhaemoglobin at 1.74 A resolution. J. Mol. Biol. 175 (2), 159–174. 10.1016/0022-2836(84)90472-8 6726807

[B10] Han Y. Yuan K. Zheng Y. Lu L. (2020). Orexin receptor antagonists as emerging treatments for psychiatric disorders. Neurosci. Bull. 36 (4), 432–448. 10.1007/s12264-019-00447-9 31782044 PMC7142186

[B11] Hertenstein E. Feige B. Gmeiner T. Kienzler C. Spiegelhalder K. Johann A. (2019). Insomnia as a predictor of mental disorders: a systematic review and meta-analysis. Sleep. Med. Rev. 43, 96–105. 10.1016/j.smrv.2018.10.006 30537570

[B12] Huang D. W. Sherman B. T. Lempicki R. A. (2009). Systematic and integrative analysis of large gene lists using DAVID bioinformatics resources. Nat. Protoc. 4 (1), 44–57. 10.1038/nprot.2008.211 19131956

[B13] Huang W. Y. Zou X. Lu F. E. Su H. Zhang C. Ren Y. L. (2018). Jiao-tai-wan up-regulates hypothalamic and peripheral circadian clock gene cryptochrome and activates PI3K/AKT signaling in partially sleep-deprived rats. Curr. Med. Sci. 38 (4), 704–713. 10.1007/s11596-018-1934-x 30128882

[B14] Hung W. Ho C. Pan M. (2020). Targeting the NLRP3 Inflammasome in Neuroinflammation: health promoting effects of dietary phytochemicals in neurological disorders. Mol. Nutr. Food Res. 64 (4), e1900550. 10.1002/mnfr.201900550 31675164

[B16] Kim S. Chen J. Cheng T. Gindulyte A. He J. He S. (2023). PubChem 2023 update. Nucleic Acids Res. 51 (1), D1373–D1380. 10.1093/nar/gkac956 36305812 PMC9825602

[B17] Kim S. Jo K. Hong K. B. Han S. H. Suh H. J. (2019). GABA and l-theanine mixture decreases sleep latency and improves NREM sleep. Pharm. Biol. 57 (1), 65–73. 10.1080/13880209.2018.1557698 30707852 PMC6366437

[B18] Lauss M. Kriegner A. Vierlinger K. Noehammer C. (2007). Characterization of the drugged human genome. Pharmacogenomics 8 (8), 1063–1073. 10.2217/14622416.8.8.1063 17716238

[B19] Levenga J. Wong H. Milstead R. A. Keller B. N. Laplante L. E. Hoeffer C. A. (2017). AKT isoforms have distinct hippocampal expression and roles in synaptic plasticity. eLife 6, e30640. 10.7554/eLife.30640 29173281 PMC5722612

[B21] Li Z. H. Cheng L. Wen C. Ding L. You Q. Y. Zhang S. B. (2022). Activation of CNR1/PI3K/AKT pathway by tanshinone IIA protects hippocampal neurons and ameliorates sleep deprivation-induced cognitive dysfunction in rats. Front. Pharmacol. 13, 823732. 10.3389/fphar.2022.823732 35295327 PMC8920044

[B24] Lin J. Yao K. Wang Q. Hua X. (2021). Mechanism of Xuefu Zhuyu Decoction in treatment of mycocardial infraction based on network pharmacology and molecular docking. China J. Chin. Materia Medica 46 (04), 885–893. 10.19540/j.cnki.cjcmm.20201106.402 33645093

[B26] Liu Y. Pan J. Sun H. Wang G. Wang W. Wu H. (2019). “Guidelines for the diagnosis and treatment of insomnia disorders in China,” in The first annual academic conference of the northeast sleep working committee of the China sleep research association and the second annual academic conference of the sleep branch of the heilongjiang association of integrative medicine (Harbin, Heilongjiang, China: Henan University of Chinese Medicine), 10.

[B27] Lu Y. Wang C. Xue Z. Li C. Zhang J. Zhao X. (2014). PI3K/AKT/mTOR signaling-mediated neuropeptide VGF in the hippocampus of mice is involved in the rapid onset antidepressant-like effects of GLYX-13. Int. J. Neuropsychopharmacol. 18 (5), pyu110. 10.1093/ijnp/pyu110 25542689 PMC4376553

[B28] Luo B. H. Pang Y. Z. Zhang L. Wu L. B. He J. Li W. K. (2020). Mechanism study on the acupuncture treatment of PCPA insomnia through the post-receptor signal pathway of 5-HT1A and 5-HT2A. J. Guangxi Univ. Nat. Sci. Ed. 45 (05), 1211–1216. 10.13624/j.cnki.issn.1001-7445.2020.1211

[B29] Meng H. Gong J. Fang L. Song Z. Wu F. Zhou B. (2013). Effect of interferon-γ on NF-κB and cytokine IL-18 and IL-27 in acute pancreatitis. Bosn. J. Basic Med. Sci. 13 (2), 114–118. 10.17305/bjbms.2013.2391 23725508 PMC4333930

[B30] Motivala S. J. (2011). Sleep and inflammation: psychoneuroimmunology in the context of cardiovascular disease. Ann. Behav. Med. a Publ. Soc. Behav. Med. 42 (2), 141–152. 10.1007/s12160-011-9280-2 21604067

[B31] Najafian B. Alpers C. E. Fogo A. B. (2011). Pathology of human diabetic nephropathy. Contrib. Nephrol. 170, 36–47. 10.1159/000324942 21659756

[B32] Paul S. Vidusha K. Thilagar S. Lakshmanan D. K. Ravichandran G. Arunachalam A. (2022). Advancement in the contemporary clinical diagnosis and treatment strategies of insomnia disorder. Sleep. Med. 91, 124–140. 10.1016/j.sleep.2022.02.018 35305527

[B33] Pinero J. Sauch J. Sanz F. Furlong L. I. (2021). The DisGeNET cytoscape app: exploring and visualizing disease genomics data. Comp. Struct. Biotechnol. J. 19, 2960–2967. 10.1016/j.csbj.2021.05.015 PMC8163863 34136095

[B34] Punnoose A. R. Golub R. M. Burke A. E. (2012). JAMA patient page. Insomnia. JAMA-J. Am. Med. Assoc. 307 (24), 2653. 10.1001/jama.2012.6219 22735439

[B36] Ru J. Li P. Wang J. Zhou W. Li B. Huang C. (2014). Tcmsp: a database of systems pharmacology for drug discovery from herbal medicines. J. Cheminformatics. 6, 13. 10.1186/1758-2946-6-13 PMC4001360 24735618

[B37] Sakurai T. Amemiya A. Ishii M. Matsuzaki I. Chemelli R. M. Tanaka H. (1998). Orexins and orexin receptors: a family of hypothalamic neuropeptides and G protein-coupled receptors that regulate feeding behavior. Cell 92 (4), 573–585. 10.1016/s0092-8674(00)80949-6 9491897

[B38] Shang Z. J. Zhang Z. Li H. (2021). Clinical analysis of drug for chronic insomnia. China J. Health Psychol. 29 (02), 197–200. 10.13342/j.cnki.cjhp.2021.02.009

[B39] Sondell M. Lundborg G. Kanje M. (1999). Vascular endothelial growth factor has neurotrophic activity and stimulates axonal outgrowth, enhancing cell survival and Schwann cell proliferation in the peripheral nervous system. J. Neurosci. 19 (14), 5731–5740. 10.1523/JNEUROSCI.19-14-05731.1999 10407014 PMC6783109

[B40] Stelzer G. Rosen N. Plaschkes I. Zimmerman S. Twik M. Fishilevich S. (2016). The GeneCards suite: from gene data mining to disease genome sequence analyses. Curr. Protoc. Bioinforma. 54, 1.30.1–1.30.33. 10.1002/cpbi.5 27322403

[B41] Su S. Y. Hsieh C. L. Wu S. L. Cheng W. Y. Li C. C. Lo H. Y. (2009). Transcriptomic analysis of EGb 761-regulated neuroactive receptor pathway *in vivo* . J. Ethnopharmacol. 123 (1), 68–73. 10.1016/j.jep.2009.02.027 19429342

[B42] Sun Y. Jin K. Xie L. Childs J. Mao X. O. Logvinova A. (2003). VEGF-induced neuroprotection, neurogenesis, and angiogenesis after focal cerebral ischemia. J. Clin. investigation 111 (12), 1843–1851. 10.1172/JCI17977 PMC161428 12813020

[B43] Szklarczyk D. Gable A. L. Lyon D. Junge A. Wyder S. Huerta-Cepas J. (2019). STRING v11: protein-protein association networks with increased coverage, supporting functional discovery in genome-wide experimental datasets. Nucleic Acids Res. 47 (1), D607–D613. 10.1093/nar/gky1131 30476243 PMC6323986

[B45] Trott O. Olson A. J. (2010). AutoDock Vina: improving the speed and accuracy of docking with a new scoring function, efficient optimization, and multithreading. J. Comput. Chem. 31 (2), 455–461. 10.1002/jcc.21334 19499576 PMC3041641

[B46] UniProt Consortium (2023). UniProt: the universal protein knowledgebase in 2023. Nucleic Acids Res. 51 (1), D523–D531. 10.1093/nar/gkac1052 36408920 PMC9825514

[B47] Wan Y. Gao W. Zhou K. Liu X. Jiang W. Xue R. (2022). Role of IGF-1 in neuroinflammation and cognition deficits induced by sleep deprivation. Neurosci. Lett. 776, 136575. 10.1016/j.neulet.2022.136575 35276231

[B48] Wang J. Wang Y. Han Z. Chen J. (2022). Clinical effect of modified Suanzaoren decoction in the treatment of insomnia. Clin. Res. Pract. 7 (17), 147–150. 10.19347/j.cnki.2096-1413.202217040

[B49] Wang Y. H. Liang S. A. Xu D. S. Lin X. A. He C. Y. Feng Y. (2011). Effect and mechanism of senkyunolide I as an anti-migraine compound from Ligusticum chuanxiong. J. Pharm. Pharmacol. 63 (2), 261–266. 10.1111/j.2042-7158.2010.01191.x 21235591

[B50] Wei Y. L. Lu F. Q. Huang X. (2019). Research progress in the diagnosis and treatment of insomnia by Chinese and Western medicine. World J. Sleep Med. 6 (04), 523–528.

[B51] Wickwire E. M. Shaya F. T. Scharf S. M. (2016). Health economics of insomnia treatments: the return on investment for a good night's sleep. Sleep. Med. Rev. 30, 72–82. 10.1016/j.smrv.2015.11.004 26874067

[B52] Wishart D. S. Feunang Y. D. Guo A. C. Lo E. J. Marcu A. Grant J. R. (2018). DrugBank 5.0: a major update to the DrugBank database for 2018. Nucleic Acids Res. 46 (D1), D1074–D1082. 10.1093/nar/gkx1037 29126136 PMC5753335

[B53] Xia L. Chen G. H. Li Z. H. Jiang S. Shen J. (2013). Alterations in hypothalamus-pituitary-adrenal/thyroid axes and gonadotropin-releasing hormone in the patients with primary insomnia: a clinical research. PLoS One 8 (8), e71065. 10.1371/journal.pone.0071065 23951080 PMC3739817

[B54] Xing X. Guo S. Zhang G. Liu Y. Bi S. Wang X. (2020). miR-26a-5p protects against myocardial ischemia/reperfusion injury by regulating the PTEN/PI3K/AKT signaling pathway. Braz. J. Med. Biol. Res. 53 (2), e9106. 10.1590/1414-431X20199106 31994603 PMC6984371

[B55] Xiong Y. K. Lin X. Liang S. Hong Y. L. Shen L. Feng Y. (2013). Identification of senkyunolide I metabolites in rats using ultra performance liquid chromatography/quadrupole-time-of-flight tandem mass spectrometry. J. Pharm. Biomed. Anal. 81-82, 178–186. 10.1016/j.jpba.2013.04.012 23666254

[B57] Yang L. Huang J. Deng L. (2023). Research progress of pharmacological actions of jujuboside A. Acad. J. Shanghai Univ. Traditional Chin. Med. 37 (01), 90–97. 10.16306/j.1008-861x.2023.01.013

[B58] Yao C. Wang Z. Jiang H. Yan R. Huang Q. Wang Y. (2021). Ganoderma lucidum promotes sleep through a gut microbiota-dependent and serotonin-involved pathway in mice. Sci. Rep. 11 (1), 13660. 10.1038/s41598-021-92913-6 34211003 PMC8249598

[B61] Zeng J. Li X. Cheng Y. Ke B. Wang R. (2019). Activation of cannabinoid receptor type 2 reduces lung ischemia reperfusion injury through PI3K/Akt pathway. Int. J. Clin. Exp. Pathol. 12 (11), 4096–4105. 31933805 PMC6949786

[B63] Zhang X. Y. Wang S. Tang L. P. Gong X. X. Pan S. M. Ding L. (2022). Research progress on suanzaoren decoction in treatment of insomnia and its mechanism. Chin. Archives Traditional Chin. Med. 40 (10), 1–7. 10.13193/j.issn.1673-7717.2022.10.001

[B64] Zhang Y. Shu Q. Liu J. F. (2021). Exploring the molecular mechanism of Zuogui pill in improving cognitive impairment caused by sleep deprivation in rats based on PI3K/AKT signaling pathway. Clin. Res. Pract. 6 (34), 6–9. 10.19347/j.cnki.2096-1413.202134002

[B65] Zhou X. M. Yin Y. J. Chang Z. Liu C. Y. Deng H. Liang B. Y. (2022). Regulation effect of Xiaoyao powder on PI3K/AKT signaling pathway in hippocampal CA1 region of CUMS rats. Acta Chin. Med. Pharmacol. 50 (01), 12–17. 10.19664/j.cnki.1002-2392.220004

[B66] Zhou Y. Zhou B. Pache L. Chang M. Khodabakhshi A. H. Tanaseichuk O. (2019). Metascape provides a biologist-oriented resource for the analysis of systems-level datasets. Nat. Commun. 10 (1), 1523. 10.1038/s41467-019-09234-6 30944313 PMC6447622

