# Supplementary material for: Exploring the mechanism of Suanzaoren decoction in treatment of insomnia based on network pharmacology and molecular docking
Source: Front Pharmacol. 2023 Aug 21;14:1145532. doi: 10.3389/fphar.2023.1145532 (PMC10475534; doi:10.3389/fphar.2023.1145532)
Supplement: Supplementary file 1 [file Table1.docx]

Table 1: Targets in PPI network.

| No. | Protein name | STRING ID |
| --- | --- | --- |
| 1 | GSR | 9606.ENSP00000221130 |
| 2 | SOD1 | 9606.ENSP00000270142 |
| 3 | CASP3 | 9606.ENSP00000311032 |
| 4 | NR1I2 | 9606.ENSP00000336528 |
| 5 | CYP3A4 | 9606.ENSP00000337915 |
| 6 | UGT1A1 | 9606.ENSP00000362549 |
| 7 | GSTP1 | 9606.ENSP00000381607 |
| 8 | TNF | 9606.ENSP00000398698 |
| 9 | AKT1 | 9606.ENSP00000451828 |
| 10 | GSTM1 | 9606.ENSP00000311469 |
| 11 | CYP1A2 | 9606.ENSP00000342007 |
| 12 | CES1 | 9606.ENSP00000353720 |
| 13 | NR1I3 | 9606.ENSP00000356959 |
| 14 | CYP1A1 | 9606.ENSP00000369050 |
| 15 | CAT | 9606.ENSP00000241052 |
| 16 | SLC6A4 | 9606.ENSP00000261707 |
| 17 | IL1B | 9606.ENSP00000263341 |
| 18 | SLC6A3 | 9606.ENSP00000270349 |
| 19 | GSK3B | 9606.ENSP00000324806 |
| 20 | FOS | 9606.ENSP00000306245 |
| 21 | CHRM4 | 9606.ENSP00000409378 |
| 22 | CHRM2 | 9606.ENSP00000399745 |
| 23 | PTGS2 | 9606.ENSP00000356438 |
| 24 | IL6 | 9606.ENSP00000385675 |
| 25 | CHRNA7 | 9606.ENSP00000407546 |
| 26 | CHRM1 | 9606.ENSP00000306490 |
| 27 | MAOA | 9606.ENSP00000340684 |
| 28 | MAOB | 9606.ENSP00000367309 |
| 29 | BACE1 | 9606.ENSP00000318585 |
| 30 | MPO | 9606.ENSP00000225275 |
| 31 | IL2 | 9606.ENSP00000226730 |
| 32 | NR3C1 | 9606.ENSP00000231509 |
| 33 | CRP | 9606.ENSP00000255030 |
| 34 | MAPK3 | 9606.ENSP00000263025 |
| 35 | STAT3 | 9606.ENSP00000264657 |
| 36 | ICAM1 | 9606.ENSP00000264832 |
| 37 | PPARG | 9606.ENSP00000287820 |
| 38 | VCAM1 | 9606.ENSP00000294728 |
| 39 | NOS3 | 9606.ENSP00000297494 |
| 40 | CXCL8 | 9606.ENSP00000306512 |
| 41 | PPARD | 9606.ENSP00000310928 |
| 42 | CD40LG | 9606.ENSP00000359663 |
| 43 | RUNX2 | 9606.ENSP00000360493 |
| 44 | MMP9 | 9606.ENSP00000361405 |
| 45 | THBD | 9606.ENSP00000366307 |
| 46 | IGFBP3 | 9606.ENSP00000370473 |
| 47 | CYP19A1 | 9606.ENSP00000379683 |
| 48 | PPARA | 9606.ENSP00000385523 |
| 49 | LDLR | 9606.ENSP00000454071 |
| 50 | IGF2 | 9606.ENSP00000391826 |
| 51 | HIF1A | 9606.ENSP00000437955 |
| 52 | ESR1 | 9606.ENSP00000405330 |
| 53 | VEGFA | 9606.ENSP00000478570 |
| 54 | IL10 | 9606.ENSP00000412237 |
| 55 | SLC6A2 | 9606.ENSP00000219833 |
| 56 | ADRA1B | 9606.ENSP00000306662 |
| 57 | ADRA1D | 9606.ENSP00000368766 |
| 58 | ADRB2 | 9606.ENSP00000305372 |
| 59 | ADRA1A | 9606.ENSP00000369960 |
| 60 | HTR3A | 9606.ENSP00000347754 |
| 61 | ADRA2C | 9606.ENSP00000386069 |
| 62 | ADRA2A | 9606.ENSP00000280155 |
| 63 | OPRD1 | 9606.ENSP00000234961 |
| 64 | CYP1B1 | 9606.ENSP00000478561 |
| 65 | ADRB1 | 9606.ENSP00000358301 |
| 66 | AR | 9606.ENSP00000363822 |
| 67 | MAPK1 | 9606.ENSP00000215832 |
| 68 | AHR | 9606.ENSP00000242057 |
| 69 | NCF1 | 9606.ENSP00000289473 |
| 70 | ACHE | 9606.ENSP00000303211 |
| 71 | CXCL10 | 9606.ENSP00000305651 |
| 72 | HSPA5 | 9606.ENSP00000324173 |
| 73 | CASP9 | 9606.ENSP00000330237 |
| 74 | PTGS1 | 9606.ENSP00000354612 |
| 75 | BCL2 | 9606.ENSP00000381185 |
| 76 | ABCC1 | 9606.ENSP00000382342 |
| 77 | ADIPOQ | 9606.ENSP00000389814 |
| 78 | HSF1 | 9606.ENSP00000431512 |
| 79 | NR3C2 | 9606.ENSP00000350815 |
| 80 | ABCC2 | 9606.ENSP00000359478 |
| 81 | CHRM3 | 9606.ENSP00000255380 |
| 82 | GABRA6 | 9606.ENSP00000274545 |
| 83 | GRIA2 | 9606.ENSP00000296526 |
| 84 | DRD1 | 9606.ENSP00000377353 |
| 85 | GABRA1 | 9606.ENSP00000393097 |
| 86 | CHRM5 | 9606.ENSP00000372750 |
| 87 | GABRA2 | 9606.ENSP00000421828 |
| 88 | GABRA5 | 9606.ENSP00000335592 |
| 89 | HTR2A | 9606.ENSP00000437737 |
| 90 | PLAT | 9606.ENSP00000220809 |
| 91 | KCNH2 | 9606.ENSP00000262186 |
| 92 | OPRM1 | 9606.ENSP00000394624 |
| 93 | ELK1 | 9606.ENSP00000483056 |
| 94 | GABRA3 | 9606.ENSP00000359337 |
| 95 | LTA4H | 9606.ENSP00000228740 |
