# Supplementary material for: Exploring the mechanism of Suanzaoren decoction in treatment of insomnia based on network pharmacology and molecular docking
Source: Front Pharmacol. 2023 Aug 21;14:1145532. doi: 10.3389/fphar.2023.1145532 (PMC10475534; doi:10.3389/fphar.2023.1145532)
Supplement: Supplementary file 2 [file Table2.docx]

Table 2: Signal pathways of KEGG analysis.

| Group ID | Term | Description | Symbls |
| --- | --- | --- | --- |
| 1_Summary | hsa05417 | Lipid and atherosclerosis | AKT1,BCL2,CASP3,CASP9,CD40LG,CYP1A1,FOS,GSK3B,HSPA5,ICAM1,IL1B,IL6,CXCL8,LDLR,MMP9,NOS3,PPARG,MAPK1,MAPK3,STAT3,TNF,VCAM1,NCF1,THBD,VEGFA,AR,ELK1,ESR1,GSTM1,GSTP1,HIF1A,IGF2,IL2,PPARD,PTGS2,PLAT,CXCL10,CAT,CHRM1,CHRM3,CHRM5,CHRNA7,GRIA2,SLC6A3,SOD1,IL10,BACE1,ABCC2,PPARA,AHR,CHRM2,OPRM1,ABCC1,OPRD1,CYP1B1,MPO,IGFBP3,ADIPOQ,RUNX2,PTGS1,NR3C2 |
| 1_Member | hsa05417 | Lipid and atherosclerosis | AKT1,BCL2,CASP3,CASP9,CD40LG,CYP1A1,FOS,GSK3B,HSPA5,ICAM1,IL1B,IL6,CXCL8,LDLR,MMP9,NOS3,PPARG,MAPK1,MAPK3,STAT3,TNF,VCAM1,NCF1 |
| 1_Member | hsa04933 | AGE-RAGE signaling pathway in diabetic complications | AKT1,BCL2,CASP3,ICAM1,IL1B,IL6,CXCL8,NOS3,MAPK1,MAPK3,STAT3,THBD,TNF,VCAM1,VEGFA |
| 1_Member | hsa05200 | Pathways in cancer | AKT1,AR,BCL2,CASP3,CASP9,ELK1,ESR1,FOS,GSK3B,GSTM1,GSTP1,HIF1A,IGF2,IL2,IL6,CXCL8,MMP9,PPARD,PPARG,MAPK1,MAPK3,PTGS2,STAT3,VEGFA |
| 1_Member | hsa05418 | Fluid shear stress and atherosclerosis | AKT1,BCL2,FOS,GSTM1,GSTP1,ICAM1,IL1B,MMP9,NOS3,PLAT,THBD,TNF,VCAM1,VEGFA,NCF1 |
| 1_Member | hsa04668 | TNF signaling pathway | AKT1,CASP3,FOS,ICAM1,IL1B,IL6,CXCL10,MMP9,MAPK1,MAPK3,PTGS2,TNF,VCAM1 |
| 1_Member | hsa04657 | IL-17 signaling pathway | CASP3,FOS,GSK3B,IL1B,IL6,CXCL8,CXCL10,MMP9,MAPK1,MAPK3,PTGS2,TNF |
| 1_Member | hsa05022 | Pathways of neurodegeneration - multiple diseases | BCL2,CASP3,CASP9,CAT,CHRM1,CHRM3,CHRM5,CHRNA7,GRIA2,GSK3B,HSPA5,IL1B,IL6,MAPK1,MAPK3,PTGS2,SLC6A3,SOD1,TNF |
| 1_Member | hsa05167 | Kaposi sarcoma-associated herpesvirus infection | AKT1,CASP3,CASP9,FOS,GSK3B,HIF1A,ICAM1,IL6,CXCL8,MAPK1,MAPK3,PTGS2,STAT3,VEGFA |
| 1_Member | hsa05161 | Hepatitis B | AKT1,BCL2,CASP3,CASP9,ELK1,FOS,IL6,CXCL8,MMP9,MAPK1,MAPK3,STAT3,TNF |
| 1_Member | hsa05163 | Human cytomegalovirus infection | AKT1,CASP3,CASP9,ELK1,GSK3B,IL1B,IL6,CXCL8,MAPK1,MAPK3,PTGS2,STAT3,TNF,VEGFA |
| 1_Member | hsa05145 | Toxoplasmosis | AKT1,BCL2,CASP3,CASP9,CD40LG,IL10,LDLR,MAPK1,MAPK3,STAT3,TNF |
| 1_Member | hsa05215 | Prostate cancer | AKT1,AR,BCL2,CASP9,GSK3B,GSTP1,MMP9,PLAT,MAPK1,MAPK3 |
| 1_Member | hsa05135 | Yersinia infection | AKT1,FOS,GSK3B,IL1B,IL2,IL6,CXCL8,IL10,MAPK1,MAPK3,TNF |
| 1_Member | hsa05142 | Chagas disease | AKT1,FOS,IL1B,IL2,IL6,CXCL8,IL10,MAPK1,MAPK3,TNF |
| 1_Member | hsa05010 | Alzheimer disease | AKT1,CASP3,CASP9,CHRM1,CHRM3,CHRM5,CHRNA7,GSK3B,IL1B,IL6,MAPK1,MAPK3,PTGS2,TNF,BACE1 |
| 1_Member | hsa01524 | Platinum drug resistance | AKT1,BCL2,CASP3,CASP9,ABCC2,GSTM1,GSTP1,MAPK1,MAPK3 |
| 1_Member | hsa05205 | Proteoglycans in cancer | AKT1,CASP3,ELK1,ESR1,HIF1A,IGF2,MMP9,MAPK1,MAPK3,STAT3,TNF,VEGFA |
| 1_Member | hsa05133 | Pertussis | CASP3,FOS,IL1B,IL6,CXCL8,IL10,MAPK1,MAPK3,TNF |
| 1_Member | hsa05140 | Leishmaniasis | ELK1,FOS,IL1B,IL10,MAPK1,MAPK3,PTGS2,TNF,NCF1 |
| 1_Member | hsa05160 | Hepatitis C | AKT1,CASP3,CASP9,GSK3B,CXCL10,LDLR,PPARA,MAPK1,MAPK3,STAT3,TNF |
| 1_Member | hsa05164 | Influenza A | AKT1,CASP3,CASP9,ICAM1,IL1B,IL6,CXCL8,CXCL10,MAPK1,MAPK3,TNF |
| 1_Member | hsa05162 | Measles | AKT1,BCL2,CASP3,CASP9,FOS,GSK3B,IL1B,IL2,IL6,STAT3 |
| 1_Member | hsa04620 | Toll-like receptor signaling pathway | AKT1,FOS,IL1B,IL6,CXCL8,CXCL10,MAPK1,MAPK3,TNF |
| 1_Member | hsa04625 | C-type lectin receptor signaling pathway | AKT1,IL1B,IL2,IL6,IL10,MAPK1,MAPK3,PTGS2,TNF |
| 1_Member | hsa04660 | T cell receptor signaling pathway | AKT1,CD40LG,FOS,GSK3B,IL2,IL10,MAPK1,MAPK3,TNF |
| 1_Member | hsa04659 | Th17 cell differentiation | AHR,FOS,HIF1A,IL1B,IL2,IL6,MAPK1,MAPK3,STAT3 |
| 1_Member | hsa04066 | HIF-1 signaling pathway | AKT1,BCL2,HIF1A,IL6,NOS3,MAPK1,MAPK3,STAT3,VEGFA |
| 1_Member | hsa04151 | PI3K-Akt signaling pathway | AKT1,BCL2,CASP9,CHRM1,CHRM2,GSK3B,IGF2,IL2,IL6,NOS3,MAPK1,MAPK3,VEGFA |
| 1_Member | hsa01521 | EGFR tyrosine kinase inhibitor resistance | AKT1,BCL2,GSK3B,IL6,MAPK1,MAPK3,STAT3,VEGFA |
| 1_Member | hsa05152 | Tuberculosis | AKT1,BCL2,CASP3,CASP9,IL1B,IL6,IL10,MAPK1,MAPK3,TNF |
| 1_Member | hsa05210 | Colorectal cancer | AKT1,BCL2,CASP3,CASP9,FOS,GSK3B,MAPK1,MAPK3 |
| 1_Member | hsa04915 | Estrogen signaling pathway | AKT1,BCL2,ESR1,FOS,MMP9,NOS3,OPRM1,MAPK1,MAPK3 |
| 1_Member | hsa04370 | VEGF signaling pathway | AKT1,CASP9,NOS3,MAPK1,MAPK3,PTGS2,VEGFA |
| 1_Member | hsa05020 | Prion disease | CASP3,CASP9,GSK3B,HSPA5,IL1B,IL6,MAPK1,MAPK3,SOD1,TNF,NCF1 |
| 1_Member | hsa04917 | Prolactin signaling pathway | AKT1,ESR1,FOS,GSK3B,MAPK1,MAPK3,STAT3 |
| 1_Member | hsa05171 | Coronavirus disease - COVID-19 | FOS,IL1B,IL2,IL6,CXCL8,CXCL10,MAPK1,MAPK3,STAT3,TNF |
| 1_Member | hsa04071 | Sphingolipid signaling pathway | AKT1,BCL2,ABCC1,NOS3,OPRD1,MAPK1,MAPK3,TNF |
| 1_Member | hsa05132 | Salmonella infection | AKT1,BCL2,CASP3,FOS,IL1B,IL6,CXCL8,MAPK1,MAPK3,TNF |
| 1_Member | hsa04380 | Osteoclast differentiation | AKT1,FOS,IL1B,PPARG,MAPK1,MAPK3,TNF,NCF1 |
| 1_Member | hsa04210 | Apoptosis | AKT1,BCL2,CASP3,CASP9,FOS,MAPK1,MAPK3,TNF |
| 1_Member | hsa05130 | Pathogenic Escherichia coli infection | CASP3,CASP9,FOS,IL1B,IL6,CXCL8,MAPK1,MAPK3,TNF |
| 1_Member | hsa05169 | Epstein-Barr virus infection | AKT1,BCL2,CASP3,CASP9,ICAM1,IL6,CXCL10,STAT3,TNF |
| 1_Member | hsa01522 | Endocrine resistance | AKT1,BCL2,ESR1,FOS,MMP9,MAPK1,MAPK3 |
| 1_Member | hsa05213 | Endometrial cancer | AKT1,CASP9,ELK1,GSK3B,MAPK1,MAPK3 |
| 1_Member | hsa04010 | MAPK signaling pathway | AKT1,CASP3,ELK1,FOS,IGF2,IL1B,MAPK1,MAPK3,TNF,VEGFA |
| 1_Member | hsa05166 | Human T-cell leukemia virus 1 infection | AKT1,ELK1,FOS,ICAM1,IL2,IL6,MAPK1,MAPK3,TNF |
| 1_Member | hsa05206 | MicroRNAs in cancer | BCL2,CASP3,CYP1B1,MMP9,ABCC1,MAPK1,MAPK3,PTGS2,STAT3,VEGFA |
| 1_Member | hsa05225 | Hepatocellular carcinoma | AKT1,ELK1,GSK3B,GSTM1,GSTP1,IGF2,MAPK1,MAPK3 |
| 1_Member | hsa05221 | Acute myeloid leukemia | AKT1,MPO,PPARD,MAPK1,MAPK3,STAT3 |
| 1_Member | hsa04935 | Growth hormone synthesis, secretion and action | AKT1,FOS,GSK3B,IGFBP3,MAPK1,MAPK3,STAT3 |
| 1_Member | hsa04919 | Thyroid hormone signaling pathway | AKT1,CASP9,ESR1,GSK3B,HIF1A,MAPK1,MAPK3 |
| 1_Member | hsa05212 | Pancreatic cancer | AKT1,CASP9,MAPK1,MAPK3,STAT3,VEGFA |
| 1_Member | hsa04062 | Chemokine signaling pathway | AKT1,GSK3B,CXCL8,CXCL10,MAPK1,MAPK3,STAT3,NCF1 |
| 1_Member | hsa04926 | Relaxin signaling pathway | AKT1,FOS,MMP9,NOS3,MAPK1,MAPK3,VEGFA |
| 1_Member | hsa05219 | Bladder cancer | CXCL8,MMP9,MAPK1,MAPK3,VEGFA |
| 1_Member | hsa04068 | FoxO signaling pathway | AKT1,CAT,IL6,IL10,MAPK1,MAPK3,STAT3 |
| 1_Member | hsa05170 | Human immunodeficiency virus 1 infection | AKT1,BCL2,CASP3,CASP9,FOS,MAPK1,MAPK3,TNF |
| 1_Member | hsa05235 | PD-L1 expression and PD-1 checkpoint pathway in cancer | AKT1,FOS,HIF1A,MAPK1,MAPK3,STAT3 |
| 1_Member | hsa05131 | Shigellosis | AKT1,BCL2,GSK3B,IL1B,CXCL8,MAPK1,MAPK3,TNF |
| 1_Member | hsa04621 | NOD-like receptor signaling pathway | BCL2,IL1B,IL6,CXCL8,MAPK1,MAPK3,TNF |
| 1_Member | hsa05211 | Renal cell carcinoma | AKT1,HIF1A,MAPK1,MAPK3,VEGFA |
| 1_Member | hsa05223 | Non-small cell lung cancer | AKT1,CASP9,MAPK1,MAPK3,STAT3 |
| 1_Member | hsa04510 | Focal adhesion | AKT1,BCL2,ELK1,GSK3B,MAPK1,MAPK3,VEGFA |
| 1_Member | hsa04662 | B cell receptor signaling pathway | AKT1,FOS,GSK3B,MAPK1,MAPK3 |
| 1_Member | hsa05224 | Breast cancer | AKT1,ESR1,FOS,GSK3B,MAPK1,MAPK3 |
| 1_Member | hsa04012 | ErbB signaling pathway | AKT1,ELK1,GSK3B,MAPK1,MAPK3 |
| 1_Member | hsa04921 | Oxytocin signaling pathway | ELK1,FOS,NOS3,MAPK1,MAPK3,PTGS2 |
| 1_Member | hsa05165 | Human papillomavirus infection | AKT1,CASP3,GSK3B,MAPK1,MAPK3,PTGS2,TNF,VEGFA |
| 1_Member | hsa04218 | Cellular senescence | AKT1,IGFBP3,IL6,CXCL8,MAPK1,MAPK3 |
| 1_Member | hsa04930 | Type II diabetes mellitus | MAPK1,MAPK3,TNF,ADIPOQ |
| 1_Member | hsa05231 | Choline metabolism in cancer | AKT1,FOS,HIF1A,MAPK1,MAPK3 |
| 1_Member | hsa04928 | Parathyroid hormone synthesis, secretion and action | BCL2,RUNX2,FOS,MAPK1,MAPK3 |
| 1_Member | hsa04722 | Neurotrophin signaling pathway | AKT1,BCL2,GSK3B,MAPK1,MAPK3 |
| 1_Member | hsa04611 | Platelet activation | AKT1,NOS3,MAPK1,MAPK3,PTGS1 |
| 1_Member | hsa04650 | Natural killer cell mediated cytotoxicity | CASP3,ICAM1,MAPK1,MAPK3,TNF |
| 1_Member | hsa04664 | Fc epsilon RI signaling pathway | AKT1,MAPK1,MAPK3,TNF |
| 1_Member | hsa05230 | Central carbon metabolism in cancer | AKT1,HIF1A,MAPK1,MAPK3 |
| 1_Member | hsa04910 | Insulin signaling pathway | AKT1,ELK1,GSK3B,MAPK1,MAPK3 |
| 1_Member | hsa04371 | Apelin signaling pathway | AKT1,NOS3,PLAT,MAPK1,MAPK3 |
| 1_Member | hsa04140 | Autophagy - animal | AKT1,BCL2,HIF1A,MAPK1,MAPK3 |
| 1_Member | hsa04550 | Signaling pathways regulating pluripotency of stem cells | AKT1,GSK3B,MAPK1,MAPK3,STAT3 |
| 1_Member | hsa04014 | Ras signaling pathway | AKT1,ELK1,IGF2,MAPK1,MAPK3,VEGFA |
| 1_Member | hsa05226 | Gastric cancer | AKT1,BCL2,GSK3B,MAPK1,MAPK3 |
| 1_Member | hsa04934 | Cushing syndrome | AHR,GSK3B,LDLR,MAPK1,MAPK3 |
| 1_Member | hsa04150 | mTOR signaling pathway | AKT1,GSK3B,MAPK1,MAPK3,TNF |
| 1_Member | hsa04658 | Th1 and Th2 cell differentiation | FOS,IL2,MAPK1,MAPK3 |
| 1_Member | hsa04960 | Aldosterone-regulated sodium reabsorption | NR3C2,MAPK1,MAPK3 |
| 1_Member | hsa05216 | Thyroid cancer | PPARG,MAPK1,MAPK3 |
| 1_Member | hsa04666 | Fc gamma R-mediated phagocytosis | AKT1,MAPK1,MAPK3,NCF1 |
| 1_Member | hsa04713 | Circadian entrainment | FOS,GRIA2,MAPK1,MAPK3 |
| 1_Member | hsa04613 | Neutrophil extracellular trap formation | AKT1,MPO,MAPK1,MAPK3,NCF1 |
| 1_Member | hsa04730 | Long-term depression | GRIA2,MAPK1,MAPK3 |
| 1_Member | hsa05168 | Herpes simplex virus 1 infection | AKT1,BCL2,CASP3,CASP9,IL1B,IL6,TNF |
| 1_Member | hsa04929 | GnRH secretion | AKT1,MAPK1,MAPK3 |
| 1_Member | hsa04072 | Phospholipase D signaling pathway | AKT1,CXCL8,MAPK1,MAPK3 |
| 1_Member | hsa04720 | Long-term potentiation | GRIA2,MAPK1,MAPK3 |
| 1_Member | hsa05218 | Melanoma | AKT1,MAPK1,MAPK3 |
| 1_Member | hsa05214 | Glioma | AKT1,MAPK1,MAPK3 |
| 1_Member | hsa05220 | Chronic myeloid leukemia | AKT1,MAPK1,MAPK3 |
| 1_Member | hsa04912 | GnRH signaling pathway | ELK1,MAPK1,MAPK3 |
| 1_Member | hsa04350 | TGF-beta signaling pathway | MAPK1,MAPK3,TNF |
| 1_Member | hsa05203 | Viral carcinogenesis | CASP3,MAPK1,MAPK3,STAT3 |
| 1_Member | hsa04916 | Melanogenesis | GSK3B,MAPK1,MAPK3 |
| 1_Member | hsa04914 | Progesterone-mediated oocyte maturation | AKT1,MAPK1,MAPK3 |
| 1_Member | hsa04015 | Rap1 signaling pathway | AKT1,MAPK1,MAPK3,VEGFA |
| 1_Member | hsa04724 | Glutamatergic synapse | GRIA2,MAPK1,MAPK3 |
| 1_Member | hsa04114 | Oocyte meiosis | AR,MAPK1,MAPK3 |
| 2_Summary | hsa05207 | Chemical carcinogenesis - receptor activation | ADRB1,ADRB2,AHR,AKT1,AR,BCL2,CHRNA7,CYP1A1,CYP1A2,CYP1B1,CYP3A4,ESR1,FOS,GSTM1,PPARA,MAPK1,MAPK3,STAT3,VEGFA,NR1I3,UGT1A1,CAT,HIF1A,SOD1,NCF1 |
| 2_Member | hsa05207 | Chemical carcinogenesis - receptor activation | ADRB1,ADRB2,AHR,AKT1,AR,BCL2,CHRNA7,CYP1A1,CYP1A2,CYP1B1,CYP3A4,ESR1,FOS,GSTM1,PPARA,MAPK1,MAPK3,STAT3,VEGFA,NR1I3,UGT1A1 |
| 2_Member | hsa05208 | Chemical carcinogenesis - reactive oxygen species | AHR,AKT1,CAT,CYP1A1,CYP1A2,CYP1B1,FOS,GSTM1,HIF1A,MAPK1,MAPK3,SOD1,VEGFA,NCF1 |
| 3_Summary | hsa04080 | Neuroactive ligand-receptor interaction | ADRA1D,ADRA1B,ADRA1A,ADRA2A,ADRA2C,ADRB1,ADRB2,CHRM1,CHRM2,CHRM3,CHRM4,CHRM5,CHRNA7,DRD1,GABRA1,GABRA2,GABRA3,GABRA5,GABRA6,GRIA2,NR3C1,HTR2A,OPRD1,OPRM1,MAPK1,MAPK3,PTGS2,HTR3A |
| 3_Member | hsa04080 | Neuroactive ligand-receptor interaction | ADRA1D,ADRA1B,ADRA1A,ADRA2A,ADRA2C,ADRB1,ADRB2,CHRM1,CHRM2,CHRM3,CHRM4,CHRM5,CHRNA7,DRD1,GABRA1,GABRA2,GABRA3,GABRA5,GABRA6,GRIA2,NR3C1,HTR2A,OPRD1,OPRM1 |
| 3_Member | hsa05033 | Nicotine addiction | CHRNA7,GABRA1,GABRA2,GABRA3,GABRA5,GABRA6,GRIA2 |
| 3_Member | hsa04723 | Retrograde endocannabinoid signaling | GABRA1,GABRA2,GABRA3,GABRA5,GABRA6,GRIA2,MAPK1,MAPK3,PTGS2 |
| 3_Member | hsa04742 | Taste transduction | CHRM3,GABRA1,GABRA2,GABRA3,GABRA5,GABRA6,HTR3A |
| 3_Member | hsa05032 | Morphine addiction | DRD1,GABRA1,GABRA2,GABRA3,GABRA5,GABRA6,OPRM1 |
| 3_Member | hsa04727 | GABAergic synapse | GABRA1,GABRA2,GABRA3,GABRA5,GABRA6 |
| 4_Summary | hsa04725 | Cholinergic synapse | ACHE,AKT1,BCL2,CHRM1,CHRM2,CHRM3,CHRM4,CHRM5,CHRNA7,FOS,MAPK1,MAPK3,ADRB1,ADRB2,DRD1,GRIA2,PPARA,HTR2A |
| 4_Member | hsa04725 | Cholinergic synapse | ACHE,AKT1,BCL2,CHRM1,CHRM2,CHRM3,CHRM4,CHRM5,CHRNA7,FOS,MAPK1,MAPK3 |
| 4_Member | hsa04024 | cAMP signaling pathway | ADRB1,ADRB2,AKT1,CHRM1,CHRM2,DRD1,FOS,GRIA2,PPARA,MAPK1,MAPK3 |
| 4_Member | hsa04810 | Regulation of actin cytoskeleton | CHRM1,CHRM2,CHRM3,CHRM4,CHRM5,MAPK1,MAPK3 |
| 4_Member | hsa04540 | Gap junction | ADRB1,DRD1,HTR2A,MAPK1,MAPK3 |
| 5_Summary | hsa04020 | Calcium signaling pathway | ADRA1D,ADRA1B,ADRA1A,ADRB1,ADRB2,CHRM1,CHRM2,CHRM3,CHRM5,CHRNA7,DRD1,HTR2A,NOS3,VEGFA,ADRA2A,ADRA2C,AKT1,OPRD1,MAPK1,MAPK3,BCL2 |
| 5_Member | hsa04020 | Calcium signaling pathway | ADRA1D,ADRA1B,ADRA1A,ADRB1,ADRB2,CHRM1,CHRM2,CHRM3,CHRM5,CHRNA7,DRD1,HTR2A,NOS3,VEGFA |
| 5_Member | hsa04022 | cGMP-PKG signaling pathway | ADRA1D,ADRA1B,ADRA1A,ADRA2A,ADRA2C,ADRB1,ADRB2,AKT1,NOS3,OPRD1,MAPK1,MAPK3 |
| 5_Member | hsa04261 | Adrenergic signaling in cardiomyocytes | ADRA1D,ADRA1B,ADRA1A,ADRB1,ADRB2,AKT1,BCL2,MAPK1,MAPK3 |
| 5_Member | hsa04970 | Salivary secretion | ADRA1D,ADRA1B,ADRA1A,ADRB1,ADRB2,CHRM3 |
| 5_Member | hsa04270 | Vascular smooth muscle contraction | ADRA1D,ADRA1B,ADRA1A,MAPK1,MAPK3 |
| 6_Summary | hsa04932 | Non-alcoholic fatty liver disease | AKT1,CASP3,FOS,GSK3B,IL1B,IL6,CXCL8,PPARA,PPARG,TNF,ADIPOQ,CD40LG,ICAM1,IL10,VCAM1,CASP9,HSF1,BCL2,PTGS2,VEGFA,ABCC2,ABCC1,IL2,STAT3,CXCL10 |
| 6_Member | hsa04932 | Non-alcoholic fatty liver disease | AKT1,CASP3,FOS,GSK3B,IL1B,IL6,CXCL8,PPARA,PPARG,TNF,ADIPOQ |
| 6_Member | hsa05144 | Malaria | CD40LG,ICAM1,IL1B,IL6,CXCL8,IL10,TNF,VCAM1 |
| 6_Member | hsa05134 | Legionellosis | CASP3,CASP9,HSF1,IL1B,IL6,CXCL8,TNF |
| 6_Member | hsa04936 | Alcoholic liver disease | AKT1,CASP3,GSK3B,IL1B,IL6,CXCL8,PPARA,TNF,ADIPOQ |
| 6_Member | hsa04064 | NF-kappa B signaling pathway | BCL2,CD40LG,ICAM1,IL1B,CXCL8,PTGS2,TNF,VCAM1 |
| 6_Member | hsa05143 | African trypanosomiasis | ICAM1,IL1B,IL6,IL10,TNF,VCAM1 |
| 6_Member | hsa05323 | Rheumatoid arthritis | FOS,ICAM1,IL1B,IL6,CXCL8,TNF,VEGFA |
| 6_Member | hsa01523 | Antifolate resistance | ABCC2,IL1B,IL6,ABCC1,TNF |
| 6_Member | hsa05321 | Inflammatory bowel disease | IL1B,IL2,IL6,IL10,STAT3,TNF |
| 6_Member | hsa04061 | Viral protein interaction with cytokine and cytokine receptor | IL2,IL6,CXCL8,IL10,CXCL10,TNF |
| 6_Member | hsa05146 | Amoebiasis | CASP3,IL1B,IL6,CXCL8,IL10,TNF |
| 6_Member | hsa04060 | Cytokine-cytokine receptor interaction | CD40LG,IL1B,IL2,IL6,CXCL8,IL10,CXCL10,TNF |
| 6_Member | hsa05332 | Graft-versus-host disease | IL1B,IL2,IL6,TNF |
| 6_Member | hsa04940 | Type I diabetes mellitus | IL1B,IL2,TNF |
| 6_Member | hsa04623 | Cytosolic DNA-sensing pathway | IL1B,IL6,CXCL10 |
| 6_Member | hsa04622 | RIG-I-like receptor signaling pathway | CXCL8,CXCL10,TNF |
| 6_Member | hsa04217 | Necroptosis | BCL2,IL1B,STAT3,TNF |
| 6_Member | hsa04640 | Hematopoietic cell lineage | IL1B,IL6,TNF |
| 7_Summary | hsa04726 | Serotonergic synapse | CASP3,HTR2A,HTR3A,MAOA,MAOB,MAPK1,MAPK3,PTGS1,PTGS2,SLC6A4 |
| 7_Member | hsa04726 | Serotonergic synapse | CASP3,HTR2A,HTR3A,MAOA,MAOB,MAPK1,MAPK3,PTGS1,PTGS2,SLC6A4 |
| 8_Summary | hsa05204 | Chemical carcinogenesis - DNA adducts | CYP1A1,CYP1A2,CYP1B1,CYP3A4,GSTM1,GSTP1,PTGS2,UGT1A1,MAOA,MAOB,CYP19A1,CES1,MPO,ABCC2,LDLR |
| 8_Member | hsa05204 | Chemical carcinogenesis - DNA adducts | CYP1A1,CYP1A2,CYP1B1,CYP3A4,GSTM1,GSTP1,PTGS2,UGT1A1 |
| 8_Member | hsa00982 | Drug metabolism - cytochrome P450 | CYP1A2,CYP3A4,GSTM1,GSTP1,MAOA,MAOB,UGT1A1 |
| 8_Member | hsa00980 | Metabolism of xenobiotics by cytochrome P450 | CYP1A1,CYP1A2,CYP1B1,CYP3A4,GSTM1,GSTP1,UGT1A1 |
| 8_Member | hsa00140 | Steroid hormone biosynthesis | CYP1A1,CYP1A2,CYP1B1,CYP3A4,CYP19A1,UGT1A1 |
| 8_Member | hsa00983 | Drug metabolism - other enzymes | CES1,CYP3A4,GSTM1,GSTP1,MPO,UGT1A1 |
| 8_Member | hsa00830 | Retinol metabolism | CYP1A1,CYP1A2,CYP3A4,UGT1A1 |
| 8_Member | hsa04976 | Bile secretion | ABCC2,CYP3A4,LDLR,UGT1A1 |
| 9_Summary | hsa00380 | Tryptophan metabolism | CAT,CYP1A1,CYP1A2,CYP1B1,MAOA,MAOB,AKT1,DRD1,FOS,GRIA2,GSK3B,SLC6A3,CASP3,CASP9,HSPA5,SOD1,MAPK1,MAPK3,NOS3 |
| 9_Member | hsa00380 | Tryptophan metabolism | CAT,CYP1A1,CYP1A2,CYP1B1,MAOA,MAOB |
| 9_Member | hsa04728 | Dopaminergic synapse | AKT1,DRD1,FOS,GRIA2,GSK3B,MAOA,MAOB,SLC6A3 |
| 9_Member | hsa05031 | Amphetamine addiction | DRD1,FOS,GRIA2,MAOA,MAOB,SLC6A3 |
| 9_Member | hsa05030 | Cocaine addiction | DRD1,GRIA2,MAOA,MAOB,SLC6A3 |
| 9_Member | hsa05012 | Parkinson disease | CASP3,CASP9,DRD1,HSPA5,MAOA,MAOB,SLC6A3,SOD1 |
| 9_Member | hsa05034 | Alcoholism | DRD1,MAOA,MAOB,MAPK1,MAPK3,SLC6A3 |
| 9_Member | hsa00330 | Arginine and proline metabolism | MAOA,MAOB,NOS3 |
| 10_Summary | hsa04931 | Insulin resistance | AKT1,GSK3B,IL6,NOS3,PPARA,STAT3,TNF,ADIPOQ,GSR,MMP9,NCF1 |
| 10_Member | hsa04931 | Insulin resistance | AKT1,GSK3B,IL6,NOS3,PPARA,STAT3,TNF |
| 10_Member | hsa04920 | Adipocytokine signaling pathway | AKT1,PPARA,STAT3,TNF,ADIPOQ |
| 10_Member | hsa05415 | Diabetic cardiomyopathy | AKT1,GSK3B,GSR,MMP9,NOS3,PPARA,NCF1 |
| 11_Summary | hsa05202 | Transcriptional misregulation in cancer | RUNX2,IGFBP3,IL6,CXCL8,MMP9,MPO,PLAT,PPARG |
| 11_Member | hsa05202 | Transcriptional misregulation in cancer | RUNX2,IGFBP3,IL6,CXCL8,MMP9,MPO,PLAT,PPARG |
| 12_Summary | hsa04913 | Ovarian steroidogenesis | CYP1A1,CYP1B1,CYP19A1,LDLR,PTGS2 |
| 12_Member | hsa04913 | Ovarian steroidogenesis | CYP1A1,CYP1B1,CYP19A1,LDLR,PTGS2 |
| 13_Summary | hsa04923 | Regulation of lipolysis in adipocytes | ADRB1,ADRB2,AKT1,PTGS1,PTGS2,LTA4H |
| 13_Member | hsa04923 | Regulation of lipolysis in adipocytes | ADRB1,ADRB2,AKT1,PTGS1,PTGS2 |
| 13_Member | hsa00590 | Arachidonic acid metabolism | LTA4H,PTGS1,PTGS2 |
| 14_Summary | hsa05330 | Allograft rejection | CD40LG,IL2,IL10,TNF,AKT1,BCL2,IL6,STAT3 |
| 14_Member | hsa05330 | Allograft rejection | CD40LG,IL2,IL10,TNF |
| 14_Member | hsa04630 | JAK-STAT signaling pathway | AKT1,BCL2,IL2,IL6,IL10,STAT3 |
| 14_Member | hsa04672 | Intestinal immune network for IgA production | CD40LG,IL2,IL6,IL10 |
| 14_Member | hsa05310 | Asthma | CD40LG,IL10,TNF |
| 14_Member | hsa05320 | Autoimmune thyroid disease | CD40LG,IL2,IL10 |
| 14_Member | hsa05322 | Systemic lupus erythematosus | CD40LG,IL10,TNF |
| 15_Summary | hsa05222 | Small cell lung cancer | AKT1,BCL2,CASP3,CASP9,PTGS2,CAT,GRIA2,HSPA5,SOD1,TNF,CD40LG,ICAM1,IGFBP3,PPARG |
| 15_Member | hsa05222 | Small cell lung cancer | AKT1,BCL2,CASP3,CASP9,PTGS2 |
| 15_Member | hsa05014 | Amyotrophic lateral sclerosis | BCL2,CASP3,CASP9,CAT,GRIA2,HSPA5,SOD1,TNF |
| 15_Member | hsa05416 | Viral myocarditis | CASP3,CASP9,CD40LG,ICAM1 |
| 15_Member | hsa04115 | p53 signaling pathway | BCL2,CASP3,CASP9,IGFBP3 |
| 15_Member | hsa04215 | Apoptosis - multiple species | BCL2,CASP3,CASP9 |
| 15_Member | hsa05016 | Huntington disease | CASP3,CASP9,GRIA2,PPARG,SOD1 |
| 16_Summary | hsa03320 | PPAR signaling pathway | PPARA,PPARD,PPARG,ADIPOQ,AKT1,CAT,ADRA1A |
| 16_Member | hsa03320 | PPAR signaling pathway | PPARA,PPARD,PPARG,ADIPOQ |
| 16_Member | hsa04211 | Longevity regulating pathway | AKT1,CAT,PPARG,ADIPOQ |
| 16_Member | hsa04152 | AMPK signaling pathway | ADRA1A,AKT1,PPARG,ADIPOQ |
| 17_Summary | hsa04670 | Leukocyte transendothelial migration | ICAM1,MMP9,VCAM1,NCF1 |
| 17_Member | hsa04670 | Leukocyte transendothelial migration | ICAM1,MMP9,VCAM1,NCF1 |
| 18_Summary | hsa00480 | Glutathione metabolism | GSR,GSTM1,GSTP1 |
| 18_Member | hsa00480 | Glutathione metabolism | GSR,GSTM1,GSTP1 |
| 19_Summary | hsa04213 | Longevity regulating pathway - multiple species | AKT1,CAT,SOD1 |
| 19_Member | hsa04213 | Longevity regulating pathway - multiple species | AKT1,CAT,SOD1 |
| 20_Summary | hsa04721 | Synaptic vesicle cycle | SLC6A2,SLC6A3,SLC6A4 |
| 20_Member | hsa04721 | Synaptic vesicle cycle | SLC6A2,SLC6A3,SLC6A4 |
